# Relative decline in serum albumin help to predict anastomotic leakage for female patients following sphincter-preserving rectal surgery

**DOI:** 10.1186/s12893-023-01923-w

**Published:** 2023-02-19

**Authors:** Kang Hu, Ke Tan, Quanzhen Shang, Chao Li, Zhe Zhang, Bin Huang, Song Zhao, Fan Li, Anping Zhang, Chunxue Li, Baohua Liu, Weidong Tong

**Affiliations:** 1 Department of General Surgery, Daping Hospital, Army Medical University, Chongqing, 400042 China; 2 Third Division Hospital, Xinjiang Production and Construction Corps, Xinjiang, China; 3 Faculty of Medicine, Autonomous University of Madrid, Madrid, Spain

**Keywords:** Sphincter-preserving rectal surgery, Anastomotic leakage, Serum albumin, Gender difference, ROC analysis

## Abstract

**Background:**

Patients with normal preoperative serum albumin still suffer from a significant reduction in serum albumin after major abdominal surgery. The current study aims to explore the predictive value of ∆ALB for AL in patients with normal serum albumin and examine whether there is a gender difference in the prediction of AL.

**Methods:**

Medical reports of consecutive patients undergoing elective sphincter-preserving rectal surgery between July 2010 and June 2016 were reviewed. Receiver operating characteristic (ROC) analysis was adopted to examine the predictive ability of ∆ALB and determine the cut-off value according to the Youden index. The logistic regression model was performed identify independent risk factors for AL.

**Results:**

Out of the 499 eligible patients, 40 experienced AL. Results of the ROC analyses showed that ΔALB displayed a significant predictive value for females, and the AUC value was 0.675 (P = 0.024), with a sensitivity of 93%. In male patients, the AUC was 0.575 (P = 0.22), but did not reach a significant level. In the multivariate analysis, ∆ALB ≥ 27.2% and low tumor location prove to be independent risk factors for AL in female patients.

**Conclusions:**

The current study suggested that there may be a gender difference in the prediction of AL and ∆ ALB can serve as a potential predictive biomarker for AL in females. A cut-off value of the relative decline in serum albumin can help predict AL in female patients as early as postoperative day 2. Although our study needs further external validation, our findings may provide an earlier, easier and cheaper biomarker for the detection of AL.

## Background

Despite surgical and perioperative management significantly improving over the last few decades, anastomotic leakage (AL) remains the most severe complication after sphincter-preserving rectal surgery [1].

[2–4] Some data suggest that preoperative hypoalbuminemia might be related to an increased risk of AL [2], while this an established risk factor for complications in general [4–7]. However, patients with normal preoperative serum albumin still suffer from a significant reduction in serum albumin after major abdominal surgery [8, 9]. Recently, several studies have demonstrated that a relative decline in serum albumin (∆ALB) is associated with clinical outcomes following surgery [10–13]. And due to a variety of advantages of ∆ALB including reflection of the inflammatory response, quicker kinetics, and easy availability [14], ∆ALB has been suggested to serve as a potential biomarker to predict postoperative complications, which may exhibit an earlier predictive ability than CRP [10]. However, at present, there is no study specifically addressing the predictive value of ∆ALB for AL after sphincter-preserving rectal surgery. Furthermore, in previous literature, we found gender may be related to ∆ALB [15], however little is known about whether gender difference affects the predictive value of biomarkers on AL.

The current study aims to explore the predictive value of ∆ALB in AL in patients with normal serum albumin and examine whether there is a gender difference in the prediction of AL.

## Material and methods

This study was approved by the Ethics Committee of Daping Hospital.

### Patients

Medical reports of consecutive patients undergoing elective sphincter-preserving rectal surgery between July 2010 and June 2016 were reviewed. The inclusion criteria included: (a) patients with histologically proven primary rectal adenocarcinoma; (b) patients aged over 18 years. The exclusion criteria included: (a) patients with the creation of a protective stoma in initial operation; (b) patients who underwent total colectomy, subtotal colectomy, or multi-visceral resection; (c) patients with Multiple primary colorectal carcinomas (MPCC); (d) patients with ongoing infection; (e) patients without available serum albumin data; (f) patients injected with albumin preoperatively or before the measurement of serum albumin within POD 2; In addition, patients with serum albumin < 35 g/L before surgery were also excluded from this study due to hypoalbuminemia might be related to AL.

### Data collection

The following data were collected from each patient: age, gender, body mass index (BMI), serum albumin value, American Society of Anesthesiology (ASA) grade, the distance of the tumor from the anal verge, anastomotic technique (stapled or handsewn), tumor staging (0–IV), preoperative chemo-radiotherapy, surgery approach (open or laparoscopic), conversion to open operation, estimated blood loss, operation time, comorbidities (hypertension, diabetes mellitus), infusion of exogenous albumin, smoking history, previous abdominal surgery.

### Primary endpoint

The primary endpoint of the current study was AL within 30 postoperative days.

### Definition

Based on previous reports [16], clinical AL in the current was defined as the presence of communication through the bowel lumen caused by a defect within the anastomosis. ALs were categorized into three types: type A which requires no additional treatment, type B which requires additional treatment other than relaparotomy, and type C which requires relaparotomy. All AL cases in the current study presented symptoms or signs including fecal or gas discharge from the pelvic drain or vagina, and peritonitis, and were confirmed by imaging or relaparotomy or by digital examination, or reoperation.

According to previously published research [10], the relative decline in serum albumin within 2 postoperative days (∆ALB) was: (preoperative serum albumin – nadir serum albumin within POD 2)/ preoperative serum albumin × 100%.

### Statistical analysis

Statistical analyses were conducted using the SPSS 25.0 and R 4.2.2 statistical software. For univariate analyses, continuous variables were assessed using the student’s t-test or the Mann–Whitney U test. Categorical variables were compared using Pearson’s chi-square or Fisher’s exact tests. Receiver operating characteristic (ROC) analysis was adopted to examine the predictive ability of ∆ALB and determine the cut-off value according to the Youden index. In order to examine the gender difference in the predictive effect of ∆ALB on AL, we conducted ROC analysis for female and male respectively. The logistic regression model was performed to identify independent risk factors for AL. Variables with a p-value of less than 0.05 in univariate analysis were selected into the logistic regression model with backward elimination. In order to examine our prediction model, bootstrap analysis with 1,000 resamples was conducted to perform internal validation using R software (version 4.2.2), and the area under curve (AUC) of the receiver operating characteristic curve (ROC) was developed to examine our prediction model. A P-value of less than 0.05 (P < 0.05) was considered statistically significant.

## Results

### Patient characteristics

**Fig. 1:**
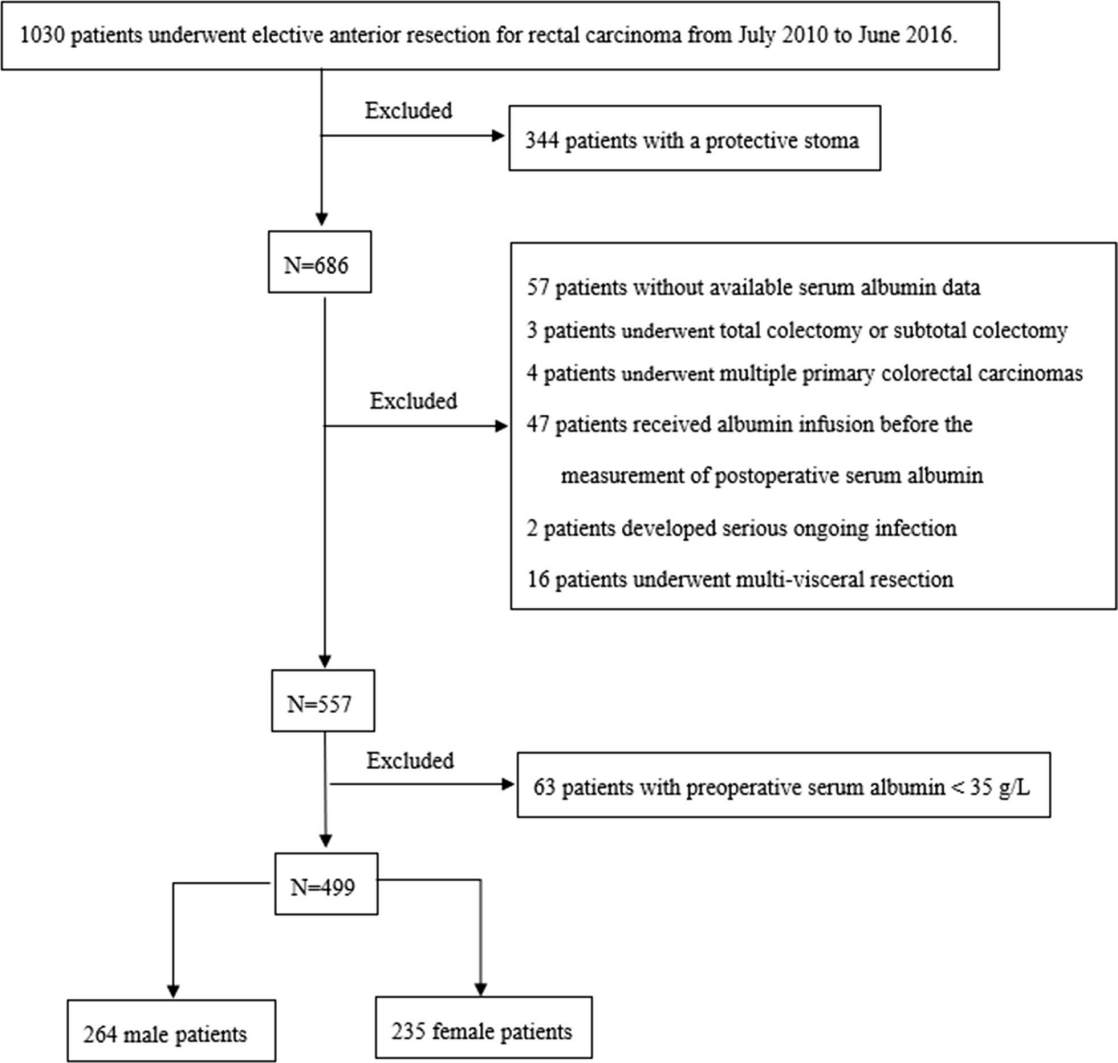
Flow chart of the patients included in the current study

A total of 499 patients (264 males and 235 females) undergoing sphincter-preserving rectal surgery without protective stoma were included in the analyses. (Fig. 1) The mean age was 58.7 years. Out of the 499 eligible patients, laparoscopic surgery was successfully performed on 413 (82.7%) patients, while 10 patients were converted from laparoscopic to open surgery. The remaining 76 patients (15.2%) underwent open surgery. Among all the patients, 40 developed AL, i.e., 8% incidence. Among the 40 patients with AL, 29 patients with type C AL were confirmed during a secondary operation, and a diverting stoma was created to improve patient symptoms. The other 11 patients (type B) were treated with medication and/or gastrointestinal decompression. Out of the 40 patients with AL, 6 patients presented clinical signs within postoperative 30 days after discharge; they were eventually confirmed after readmission. Other details were shown in Table 1.

**Table 1:** Patients characteristics

	Non-AL (n = 459)	AL (n = 40)	Total (n = 499)	P
Age (years), mean ± SD	58.6 ± 10.9	59.3 ± 10.5	58.7 ± 10.9	0.723
Gender (%)				0.205
Female	220 (47.9%)	15 (37.5%)	235 (47.1%)	
Male	239 (52.1%)	25 (62.5%)	264 (52.9%)	
BMI				0.391
< 25 kg/m^2^	344 (74.9%)	31 (77.5%)	375 (75.2%)	
≥ 25 kg/m^2^	76 (16.6%)	8 (20%)	84 (16.8%)	
Missing	39 (8.5%)	1 (2.5%)	40 (8%)	
Diabetes mellitus (%)				0.757
Yes	35 (7.6%)	2 (5%)	37 (7.4%)	
No	424 (92.4%)	38 (95%)	462 (92.6%)	
Hypertension (%)				0.337
Yes	66 (14.4%)	8 (20%)	74 (14.8%)	
No	393 (85.6%)	32 (80%)	425 (85.2%)	
Previous abdominal surgery (%)				0.772
Yes	77 (16.8%)	6 (15%)	83 (16.6%)	
No	382 (83.2%)	34 (85%)	416 (83.4%)	
Preoperative serum albumin(g/L), mean ± SD	41 ± 3.3	41.4 ± 3	41 ± 3.3	0.457
Neoadjuvant therapy (%)				1.000
Yes	9 (2%)	0 (0%)	9 (1.8%)	
No	450 (98%)	40 (100%)	490 (98.2%)	
ASA score				1.000
III–IV	22 (4.8%)	2 (5%)	24 (4.8%)	
I–II	437 (95.2%)	38 (95%)	475 (95.2%)	
Operation time (%)				0.968
< 180 min	357 (77.8%)	31 (77.5%)	388 (77.8%)	
≥ 180 min	102 (102)	9 (22.5%)	111 (22.2%)	
Tumor location (%)				0.366
≤ 5 cm	119 (25.9%)	13 (32.5%)	132 (26.5%)	
> 5 cm	340 (74.1%)	27 (67.5%)	367 (73.5%)	
AJCC stage (%)				0.605
0–II	283 (61.7%)	23 (57.5%)	306 (61.3%)	
III–IV	176 (38.3%)	17 (42.5%)	193 (38.7%)	
Type of anastomosis (%)				0.338
Stapled	429 (93.5%)	36 (90%)	465 (6.8%)	
Hand-sewn	30 (6.5%)	4 (10%)	34 (6.8%)	
Estimated blood loss (%)				0.016
≤ 300 ml	444 (96.7%)	35 (87.5%)	479 (96%)	
> 300 ml	15 (3.3%)	5 (12.5%)	20 (4%)	
Surgery approach (%)				0.851
Laparoscopic surgery	380 (82.8%)	33 (82.5%)	413 (82.8%)	
Open surgery	69 (15%)	7 (17.5%)	76 (15.2%)	
Conversion	10 (2.2%)	0 (0%)	10 (2%)	
Postoperative serum albumin (g/L), mean ± SD	29.3 ± 3.5	28.3 ± 3.2	29.2 ± 3.5	0.074
The average relative decline in serum albumin	28.2% ± 8.8%	31.5% ± 8%	28.5% ± 8.8%	0.026

*AL* anastomotic leakage, *BMI* body mass index, *AJCC* American Joint Committee on Cancer, *ASA *American Society of Anesthesiologists, *POD* postoperative day, *ALB* albumin, *∆ALB* (preoperative ALB – nadir ALB within POD 2)/preoperative ALB × 100%

As shown in Table 2, women were significantly younger than men on average (57.5 ± 11.1 vs 59.7 ± 10.6, P < 0.05) and experienced significantly more frequent previous abdominal surgery than men (22.6% vs 11.4%, P < 0.05). Furthermore, only 2 women (0.9%) reported that they smoked compared with 85 (32.2%) in men. Meanwhile, no women received neoadjuvant therapy while 9 men choose adjuvant therapy before surgery.

**Table 2:** Comparisons of clinical characteristics by gender

	Female	Male	Total	P
	(n = 235)	(n = 264)	(n = 499)	
Age (%)				0.046
≤ 65 years	175 (74.5%)	175 (66.3%)	350 (70.1%)	
> 65 years	60 (25.5%)	89 (33.7%)	149 (29.9%)	
Age (years), mean ± SD	57.5 ± 11.1	59.7 ± 10.6	58.7 ± 10.9	0.027
BMI				0.119
< 25 kg/m^2^	173 (73.6%)	202 (76.5%)	375 (75.2%)	
≥ 25 kg/m^2^	47 (20%)	37 (14%)	84 (16.8%)	
Missing	15 (6.4%)	25 (9.5%)	40 (8%)	
Smoking	2 (0.9%)	85 (32.2%)	87 (17.4%)	< 0.001
Diabetes mellitus (%)				0.844
Yes	18 (7.7%)	19 (7.2%)	37 (7.4%)	
No	217 (92.3%)	245 (92.8%)	462 (92.6%)	
Hypertension (%)				0.641
Yes	33 (14%)	41 (15.5%)	74 (14.8%)	
No	202 (86%)	223 (84.5%)	425 (85.2%)	
Previous abdominal surgery (%)				0.001
Yes	53 (22.6%)	30 (11.4%)	83 (16.6%)	
No	182 (77.4%)	234 (88.6%)	416 (83.4%)	
Preoperative serum albumin(g/L), mean ± SD	41.2 ± 3.3	40.9 ± 3.3	41 ± 3.3	0.35
Neoadjuvant therapy (%)				0.004
Yes	0 (0%)	9 (3.4%)	9 (1.8%)	
No	235 (100%)	255 (96.6%)	490 (98.2%)	
ASA score				0.166
III–IV	8 (3.4%)	16 (6.1%)	24 (4.8%)	
I–II	227 (96.6%)	248 (93.9%)	475 (95.2%)	
Operation time (%)				0.624
< 180 min	185 (78.7%)	203 (76.9%)	388 (77.8%)	
≥ 180 min	50 (21.3%)	61 (23.1%)	111 (22.2%)	
Tumor location (%)				0.564
≤ 5 cm	65 (27.7%)	67 (25.4%)	132 (26.5%)	
> 5 cm	170 (72.3%)	197 (74.6%)	367 (73.5%)	
AJCC stage (%)				0.063
0–II	134 (57%)	172(65.2%)	306(61.3%)	
III–IV	101 (43%)	92(34.8%)	193(38.7%)	
Type of anastomosis (%)				0.284
Stapled	222 (94.9%)	243 (97%)	465 (6.8%)	
Hand-sewn	13 (5.1%)	21 (3%)	34 (6.8%)	
Estimated blood loss (%)				0.238
≤ 300 ml	223 (96.7%)	256 (87.5%)	479 (96%)	
> 300 ml	12 (3.3%)	8 (12.5%)	20 (4%)	
Surgery approach (%)				0.494
Laparoscopic surgery	190 (80.9%)	223 (84.5%)	413 (82.8%)	
Open surgery	39 (16.6%)	37 (14%)	76 (15.2%)	
Conversion	6 (2.6%)	4 (1.5%)	10 (2%)	
Postoperative serum albumin (g/L), mean ± SD	28.9 ± 3.5	29.5 ± 3.5	29.3 ± 3.5	0.057
The average relative decline in serum albumin	29.5% ± 8.8%	27.6% ± 8.7%	28.5% ± 8.8%	0.015
Anastomotic leakage	15 (6.4%)	25 (9.5%)	40 (8%)	0.205

*BMI* body mass index, *AJCC* American Joint Committee on Cancer, *ASA* American Society of Anesthesiologists

There were no differences between males and females in BMI, diabetes mellitus, hypertension, ASA score, operation time, tumor location, AJCC stage, type of anastomosis, estimated blood loss, or surgery approach. Of note, AL occurred more frequently in male patients than female patients although the difference did not reach significant significance (9.5% vs 6.4%, P = 0.205).

**Fig. 2:**
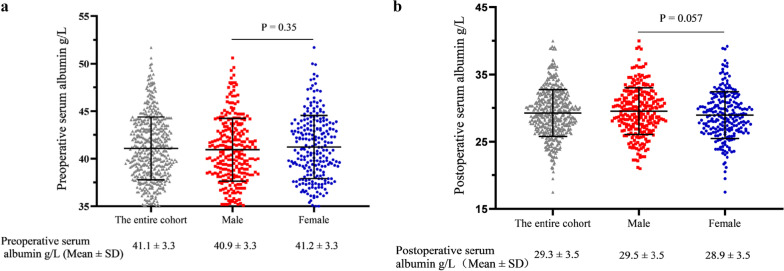
**a** inllustrates there was no significant difference in preoperative serum albumin levels between male and female patients. As shown in **b**, the results were similar when comparing postoperative serum albumin levels

**Fig. 3:**
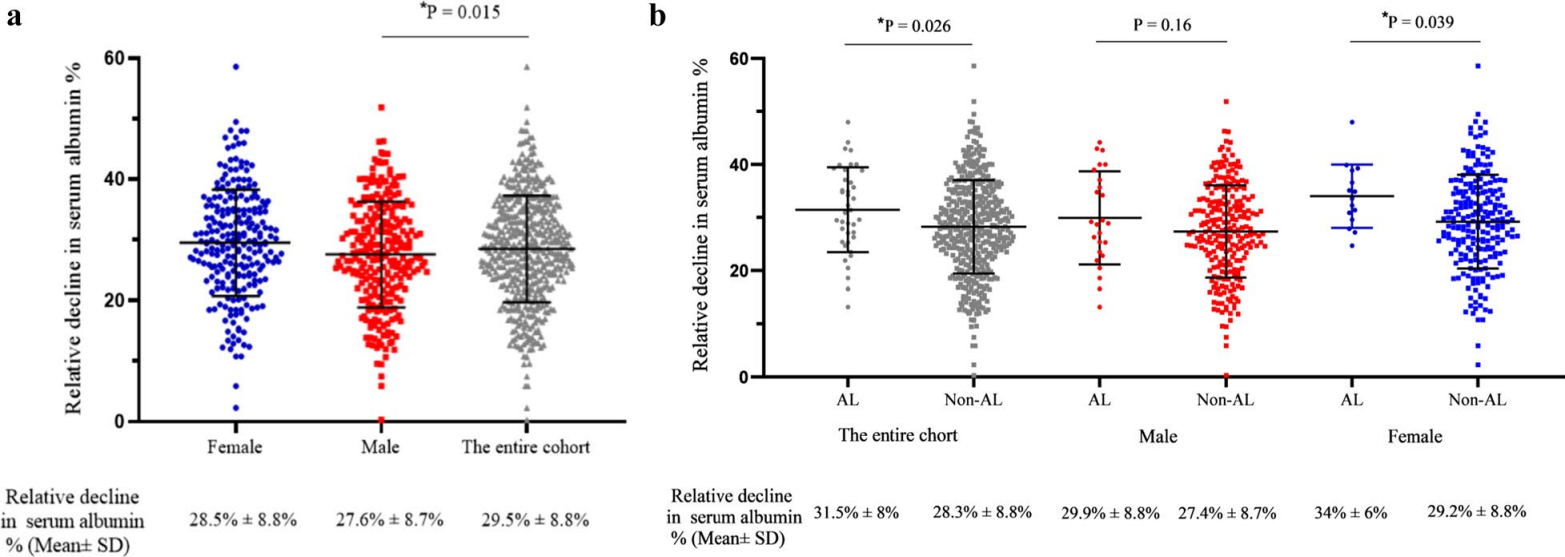
**a** shows the relative decline in serum albumin (∆ALB) in female patients was significantly higher than in male patients. In **b**, patients with AL were subject to significantly higher ∆ALB than patients without AL in the entire cohort. When grouped by gender, a significant difference in ∆ALB between patients with and without anastomotic leakage (AL) was only observed in female patients

In terms of serum albumin, Fig. 2a shows there was no significant difference in preoperative serum albumin levels between male and female patients. In Fig. 2b, the result was similar when comparing postoperative preoperative serum albumin levels. The relative decline in serum albumin (ΔALB) in female patients was significantly higher than in male patients (Fig. 3a). As displayed in Fig. 3b, in the entire cohort, patients with AL presented higher ∆ALB than patients without AL. However, when we stratified the entire cohort by gender, a significant difference in ΔALB between patients with and without AL was only observed in female patients.

### Receiver operating characteristic (ROC) curve analysis

**Fig. 4:**
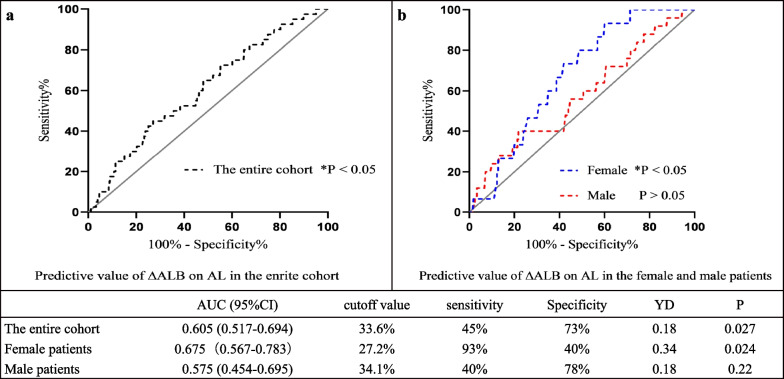
As demonstrated in **a**, ROC analysis of ∆ALB on AL only produced an AUC of 0.605 with a sensitivity of 45%. In **b**, after stratification according to gender, in female patients, ∆ALB presented a better predictive value with an AUC of and a sensitivity of 93%, whereas in male patients ∆ALB failed to prove a significant predictive value

As shown in Fig. 4a, for the entire cohort, the area under the curve (AUC) of ΔALB was 0.605 (95% CI, 0.517–0.694, P = 0.027) with a Youden index of 0.18 and a cut-off value of 33.6%. This resulted in a sensitivity of 45% and a specificity of 73%. The predictive value of ΔALB on AL was further analyzed for males and females respectively in Fig. 4b. In male patients, the AUC was 0.575 (95% CI, 0.4254–0.695) with a p-value of 0.22, did not reach a significant level. In contrast to the male, ΔALB displayed a significant predictive value in AL for females, and the AUC value was 0.675 (95% CI, 0.567–0.783, P = 0.024), with a Youden index of 0.34 and a cut-off value of 27.2%, a sensitivity of 93%, a specificity of 40%.

### Independent risk factors for AL in female patients

As shown in Table 3, a univariate analysis was conducted to compare characteristics between patients with and without AL in female patients. Female patients with AL were tend to be subject to more estimated blood loss. Patients with an ∆ALB ≥ 27.2% or a lower tumor location or a hand-sewn anastomosis were more prone to AL. In the multivariate analysis, ∆ALB ≥ 27.2% and low tumor location prove to be independent risk factors for AL in female patients (Table 4).

**Table 3:** Comparisons of patients with and without AL in female patients

	Non-AL (n = 220)	AL (n = 15)	Total (n = 235)	P
Age (%)				0.766
≤ 65 years	163 (74.1%)	12 (80%)	175 (74.5%)	
> 65 years	57 (25.9%)	3 (20%)	60 (25.5%)	
BMI				0.560
< 25 kg/m^2^	162 (73.6%)	11 (73.3%)	173 (73.6%)	
≥ 25 kg/m^2^	43 (19.5%)	4 (26.7%)	47 (20%)	
Missing	15 (6.8%)	0 (0%)	15 (6.4%)	
Diabetes mellitus (%)				0.613
Yes	18 (8.2%)	0 (0%)	18 (7.7%)	
No	202 (91.8%)	15 (100%)	217 (92.3%)	
Hypertension (%)				1.000
Yes	31 (14.1%)	2 (13.3%)	33 (14%)	
No	189 (85.9%)	13 (86.7%)	202 (86%)	
Previous abdominal surgery (%)				1.000
Yes	50 (22.7%)	3 (20%)	53 (22.6%)	
No	170 (77.3%)	12 (80%)	182 (77.4%)	
Preoperative serum albumin(g/L), mean ± SD	41.2 ± 3.3	42 ± 3.6	41.2 ± 3.3	0.324
ASA score				1.000
III–IV	8 (3.6%)	0 (0%)	8 (3.4%)	
I–II	212 (96.4%)	15 (100%)	227 (96.6%)	
Operation time (%)				0.323
< 180 min	175 (79.5%)	10 (66.7%)	185 (78.7%)	
≥ 180 min	45 (20.5%)	5 (33.3%)	50 (21.3%)	
Tumor location (%)				0.001
≤ 5 cm	55 (25%)	10 (66.7%)	65 (27.7%)	
> 5 cm	165 (75%)	5 (33.3%)	170 (72.3%)	
AJCC stage (%)				0.810
0–II	125 (56.8%)	9 (60%)	134 (57%)	
III–IV	95 (43.2%)	6 (40%)	101 (43%)	
Type of anastomosis (%)				0.041
Stapled	210 (94.6%)	12 (80%)	222 (94.5%)	
Hand-sewn	10 (4.5%)	3 (20%)	13 (5.5%)	
Estimated blood loss (%)				0.033
≤ 300 ml	211 (95.9%)	12 (80%)	223 (94.9%)	
> 300 ml	9 (4.1%)	3 (20%)	12 (5.1%)	
Surgery approach (%)				0.527
Laparoscopic surgery	179 (81.4%)	11 (73.3%)	190 (80.9%)	
Open surgery	35 (15.9%)	4 (26.7%)	39 (16.6%)	
Conversion	6 (2.7%)	0 (0%)	6 (2.6%)	
Postoperative serum albumin (g/L), mean ± SD	29 ± 3.5	27.7 ± 3.4	28.9 ± 3.5	0.161
∆ALB (%)				0.010
< 27.2%	88 (40%)	1 (1.1%)	89 (37.9%)	
≥ 27.2%	132 (60%)	14 (93.3%)	146 (62.1%)	

*AL* anastomotic leakage, *BMI* body mass index, *AJCC* American Joint Committee on Cancer, *ASA *American Society of Anesthesiologists, *POD* postoperative day, *ALB* albumin, ∆ALB:(preoperative ALB – nadir ALB within POD 2)/preoperative ALB × 100%

**Table 4:** Multivariate analysis of risk factors for AL in female patients

Characteristic	OR	95% CI	P
Tumor location (≤ 5 cm / > 5 cm)	5.687	1.832–17.657	0.003
∆ALB (≥ 27.2% / < 27.2%)	8.727	1.111–68.57	0.039

### Internal validation of the prediction model

After a bootstrap analysis with 1000 resamples, the AUC was 0.803, 95% CI (0.798, 0.807), indicating that the prediction model had a good discrimination ability, which was similar to the AUC from the original dataset [0.802, 95% CI (0.674, 0.930)].

## Discussion

Our findings indicated ∆ALB was a potential predictive biomarker in the early detection of AL for female patients with normal preoperative serum albumin (≥ 35 g/L), while ∆ALB failed to exhibit a predictive ability on AL for male patients. Our results suggested that a cut-off value of 27.2% of ∆ALB as early as postoperative day 2 can help surgeons to identify female patients at high risk of AL. Our findings may provide surgeons with an earlier, easier method to detect AL, and may also help to reduce the economic burden on patients.

In clinical practice, serum albumin is widely used as an indicator of nutritional status and is closely associated with clinical outcomes [17]. Some reasearchers have found that preoperative hypoalbuminemia might be associated with AL after colorectal surgery in their research [2]. One possible explanation for the correlation between preoperative hypoalbuminemia and AL is that poor nutritional status negatively influences collagen synthesis and granuloma formation, leading to poor wound healing at the anastomosis site [10, 18]. Although the detrimental effect of preoperative hypoalbuminemia can be attenuated by active interventions [2, 19, 20], low serum albumin in the early postoperative stage is a common clinical scenario even for patients with normal serum albumin. This is because serum albumin falls greatly after major surgery primarily due to increased capillary leakage resulting from surgical stress [8, 10, 21]. Nonetheless, in patients with normal serum albumin, the effect of a perioperative decline in serum albumin on AL fails to attract enough attention from surgeons potentially since these patients are often in a well-nourished condition.

In fact, despite early postoperative low serum albumin often lasting for only a few days [9], the relative decline in serum albumin was revealed to be associated with postoperative complications. GE et al. [10] reported that patients with a 15% reduction in serum albumin within 2 days after surgery harbor a higher risk of postoperative complications. Also, Issangya et al. [12] asserted that in abdominal surgery, patients with a 14.77% reduction in serum albumin increased the risk of developing postoperative complications by nearly sevenfold. In line with previous studies, ROC analysis yielded a cutoff value of 27.2%, the multivariate analysis demonstrated that a reduction of 27.2% was a risk factor for AL in female patients with an OR of 7.95.

It is well established that there are inherent differences in physiology, psychology, hormone levels, and anatomy, lifestyles between women and men [22–24], which may lead to different treatment options and clinical outcomes. Therefore, some researchers argued that researchers needed to analyze results for males and females separately to avoid drawing incorrect conclusions [25]. Notably, in previous research, gender was shown to be associated with ∆ALB [10, 15], but currently, no data have been reported regarding whether there is a gender difference concerning the predictive value of ∆ALB.

The identified cut-off value of ∆ALB in our study was higher than those reported by previous studies. This was expected considering the following reasons. On one hand, AL incidence in the current study was lower than that of overall complications investigated in previous studies. For example, Issangya et al. [12] reported 11.61% as a cut-off value, while 45.9% of patients had adverse outcomes. Ge et al. [10] reported a cut-off value of 15% with postoperative complications occurring in 40.9% of patients. Unlike the above-mentioned studies assessing overall complication rates, we particularly focus on AL, the incidence of which was 6.4% in this study in female patients, apparently lower than the overall complication rates mentioned above. On the other hand, the severity of postoperative complications is likely related to the degree of capillary permeability [26], and all AL cases reported in this study were classified as major complications. As a consequence, it is reasonable ∆ALB following sphincter-preserving surgery for rectal cancer in the current study is relatively higher.

It has long been recognized that men are at a higher risk of developing AL. One widely accepted explanation for the discrepancy in the occurrence of AL between males and females is that the narrow and deep pelvis cavity in men adds to the surgical difficulty [27]. However, we consider there could be a biological explanation for the relatively high risk of developing AL in male patients. A recent experimental study by Marie Kjaer et al. indicated that on the 3rd postoperative day, total collagen concentration, which was closely associated with serum albumin, was significantly higher in the anastomotic wounds in the female animals [27]. Moreover, a recent study found that oestrogen hormone exposure may be related to incidence of AL [28]. Although the actual mechanism for AL remains unclear, our finding may provide some new insights into a prediction of AL since currently no data have been reported to deal with this subject.

This work has worth-mentioning limitations. First, this is a retrospective single-center study with limited sample size, especially only 15 AL in the women group. Secondly, AL of type A was excluded because it is difficult to identify this type in the absence of clinical signs. Thirdly, if the measurement of serum albumin were performed at a uniform time point, this study would present a more precise result. Finally, there is need of larger sample size to perform external invalidation to further examine our results.

## Conclusions

The current study suggested that there may be a gender difference in the prediction of AL and ∆ALB can serve as a potential predictive biomarker for AL in females. A cut-off value of the relative decline in serum albumin can help predict AL in female patients as early as postoperative day 2. Although our study needs further external validation, our findings may provide an earlier, easier and cheaper biomarker for the detection of AL.

## Data Availability

The data used in the current study can be obtained on request from our corresponding author.

## References

[1] Bostrom P, Haapamaki MM, Rutegard J, Matthiessen P, Rutegard M (2019). Population-based cohort study of the impact on postoperative mortality of anastomotic leakage after anterior resection for rectal cancer. BJS Open.

[2] Suding P, Jensen E, Abramson MA, Itani K, Wilson SE (2008). Definitive risk factors for anastomotic leaks in elective open colorectal resection. Arch Surg (Chicago. 1960).

[3] McDermott FD, Heeney A, Kelly ME, Steele RJ, Carlson GL, Winter DC (2015). Systematic review of preoperative, intraoperative and postoperative risk factors for colorectal anastomotic leaks. Br J Surg.

[4] Hardt J, Pilz L, Magdeburg J, Kienle P, Post S, Magdeburg R (2017). Preoperative hypoalbuminemia is an independent risk factor for increased high-grade morbidity after elective rectal cancer resection. Int J Colorectal Dis.

[5] Curran S, Apruzzese P, Kendall MC, De Oliveira G (2022). The impact of hypoalbuminemia on postoperative outcomes after outpatient surgery: a national analysis of the NSQIP database. Can J Anesth.

[6] Hu WH, Eisenstein S, Parry L, Ramamoorthy S (2019). Preoperative malnutrition with mild hypoalbuminemia associated with postoperative mortality and morbidity of colorectal cancer: a propensity score matching study. Nutr J.

[7] Meyer CP, Rios-Diaz AJ, Dalela D, Ravi P, Sood A, Hanske J (2017). The association of hypoalbuminemia with early perioperative outcomes—a comprehensive assessment across 16 major procedures. Am J Surg.

[8] Norberg Å, Rooyackers O, Segersvärd R, Wernerman J (2016). Leakage of albumin in major abdominal surgery. Crit Care (London, England).

[9] Yuan XMM, Zhang CMD, He YMD, Yuan YBM, Cai SMD, Luo NBM (2008). Is albumin administration beneficial in early stage of postoperative hypoalbuminemia following gastrointestinal surgery?: a prospective randomized controlled trial. Am J Surg.

[10] Ge X, Dai X, Ding C, Tian H, Yang J, Gong J (2017). Early postoperative decrease of serum albumin predicts surgical outcome in patients undergoing colorectal resection. Dis Colon Rectum.

[11] Li P, Li J, Lai Y, Wang Y, Wang X, Su J (2018). Perioperative changes of serum albumin are a predictor of postoperative pulmonary complications in lung cancer patients: a retrospective cohort study. J Thorac Dis.

[12] Issangya CE, Msuya D, Chilonga K, Herman A, Shao E, Shirima F (2020). Perioperative serum albumin as a predictor of adverse outcomes in abdominal surgery: prospective cohort hospital based study in Northern Tanzania. BMC Surg.

[13] Muller C, Stift A, Argeny S, Bergmann M, Gnant M, Marolt S (2018). Delta albumin is a better prognostic marker for complications following laparoscopic intestinal resection for Crohn's disease than albumin alone—a retrospective cohort study. PLoS ONE.

[14] Hubner M, Mantziari S, Demartines N, Pralong F, Coti-Bertrand P, Schafer M (2016). postoperative albumin drop is a marker for surgical stress and a predictor for clinical outcome: a pilot study. Gastroent Res Pract.

[15] Hu K, Tong W (2022). Does sex-difference matter for the decrease in serum albumin?. Dis Colon Rectum.

[16] Rahbari NN, Weitz J, Hohenberger W, Heald RJ, Moran B, Ulrich A (2010). Definition and grading of anastomotic leakage following anterior resection of the rectum: a proposal by the International Study Group of Rectal Cancer. Surgery.

[17] Almasaudi AS, Dolan RD, Edwards CA, McMillan DC (2020). Hypoalbuminemia reflects nutritional risk, body composition and systemic inflammation and is independently associated with survival in patients with colorectal cancer. Cancers..

[18] Liu X, Wu X, Zhou C, Hu T, Ke J, Chen Y (2017). Preoperative hypoalbuminemia is associated with an increased risk for intra-abdominal septic complications after primary anastomosis for Crohn’s disease. Gastroenterol Rep.

[19] Moghadamyeghaneh Z, Hwang G, Hanna MH, Phelan MJ, Carmichael JC, Mills SD (2015). Even modest hypoalbuminemia affects outcomes of colorectal surgery patients. Am J Surg.

[20] Xu H, Kong F (2020). Malnutrition-related factors increased the risk of anastomotic leak for rectal cancer patients undergoing surgery. Biomed Res Int.

[21] Labgaa I, Joliat G, Kefleyesus A, Mantziari S, Schäfer M, Demartines N (2017). Is postoperative decrease of serum albumin an early predictor of complications after major abdominal surgery? A prospective cohort study in a European centre. BMJ Open.

[22] Legato MJ, Johnson PA, Manson JE (2016). Consideration of sex differences in medicine to improve health care and patient outcomes. JAMA-J Am Med Assoc.

[23] Avery E, Clark J (2016). Sex-related reporting in randomised controlled trials in medical journals. Lancet.

[24] Katzenstein J, Steinert R, Ptok H, Otto R, Gastinger I, Lippert H (2018). Gender-specific differences of the early postoperative and oncosurgical long-term outcome in rectal cancer-data obtained in a prospective multicenter observational study. Chirurg.

[25] Clayton JA, Tannenbaum C (2016). Reporting sex, gender, or both in clinical research?. JAMA-J Am Med Assoc.

[26] Ryan AM, Hearty A, Prichard RS, Cunningham A, Rowley SP, Reynolds JV (2007). Association of hypoalbuminemia on the first postoperative day and complications following esophagectomy. J Gastrointest Surg.

[27] Kjaer M, Kristjánsdóttir H, Andersen L, Heegaard A, Ågren MS, Jorgensen LN (2018). The effect of gender on early colonic anastomotic wound healing. Int J Colorectal Dis.

[28] Rutegard M, Moshtaghi-Svensson J, Weibull CE, Ottander U, Nordenvall C, Sund M (2022). Exposure to oestrogen and risk of anastomotic leakage after colorectal cancer surgery—a clue to the different leak rates in men and women. Colorectal Dis..

